# Supplementary material for: TGF-β inhibitor treatment of H₂O₂-induced cystitis models provides biochemical mechanism for elucidating interstitial cystitis/painful bladder syndrome patients
Source: PLoS One. 2023 Nov 6;18(11):e0293983. doi: 10.1371/journal.pone.0293983 (PMC10627456; doi:10.1371/journal.pone.0293983)
Supplement: S1 Fig — The surface area is made by fluid drops of known volume (1 μL, 2.5μL, 5 μL, 10 μL, 20 μL, 50 μL, 100 μL, 150 μL, 200 μL, 250 μL, 300 μL, 350 μL, 400 μL, 500 μL). Linear correlation between liquid volume and stained area on the filter paper (r2 = 0.9924, y = 0.1401x - 20.766). (DOCX) [file pone.0293983.s001.docx]

**S1 Fig.**

**
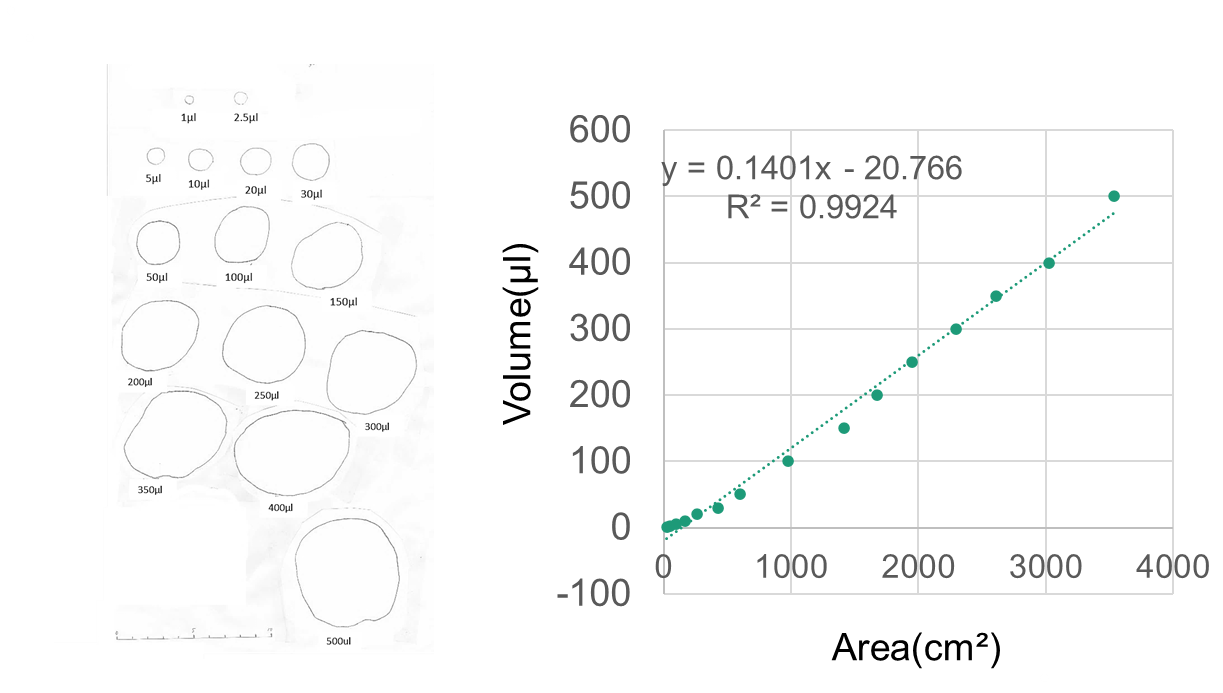
**

**S1 Fig.　Method of Voided Stain On Paper (VSOP) is shown.**The surface area is made by fluid drops of known volume (1 μL, 2.5μL, 5 μL, 10 μL, 20 μL, 50 μL, 100 μL, 150 μL, 200 μL, 250 μL, 300 μL, 350 μL, 400 μL, 500 μL). Linear correlation between liquid volume and stained area on the filter paper (r2= 0.9924, y = 0.1401x - 20.766).
