# Supplementary material for: TGF-β inhibitor treatment of H₂O₂-induced cystitis models provides biochemical mechanism for elucidating interstitial cystitis/painful bladder syndrome patients
Source: PLoS One. 2023 Nov 6;18(11):e0293983. doi: 10.1371/journal.pone.0293983 (PMC10627456; doi:10.1371/journal.pone.0293983)
Supplement: S2 Fig — Effect of intravesical infusion of H₂O₂ on the mRNA expression levels of inflammatory cytokines. The mRNA expression levels of IL1β and TGF-β1,2 in control, saline, H₂O₂ groups (respectively n = 3) were measured by real-time RT-qPCR. Each mRNA level was normalized to the β-actin mRNA level and expressed relative to the control (fold). Results are represented as means ± sd. *P < 0.05, **P < 0.01. (DOCX) [file pone.0293983.s002.docx]

**S2 Fig.**

**
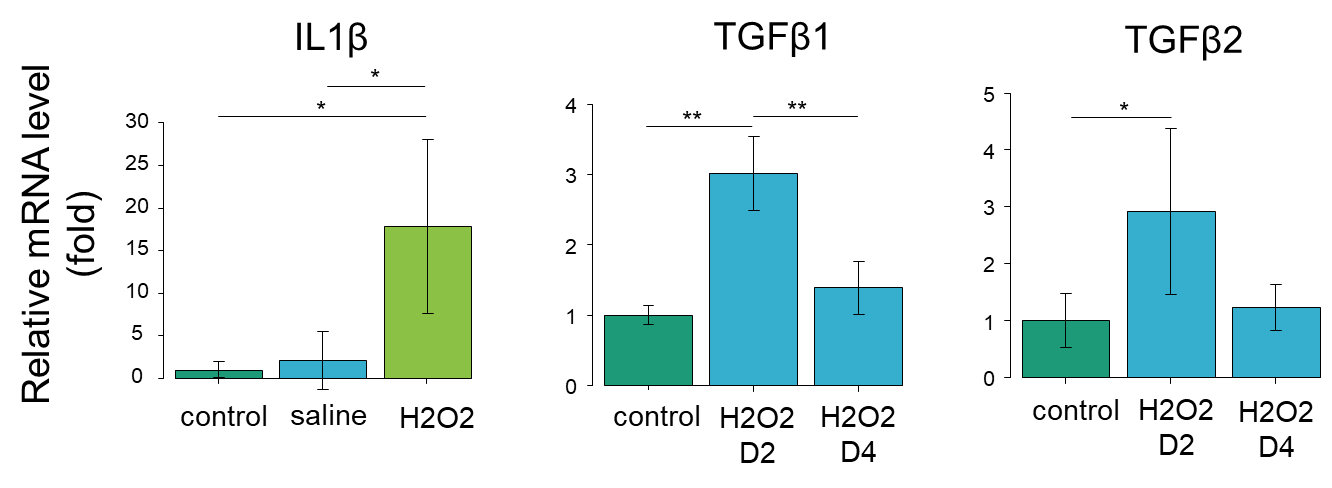
**

**S2 Fig. mRNA expressions of TGF-β are elevated in H₂O₂-induced mice.**

Effect of intravesical infusion of H₂O₂ on the mRNA expression levels of inflammatory cytokines. The mRNA expression levels of IL1β and TGF-β1,2 in control, saline, H₂O₂ groups (respectively n=3) were measured by real-time RT-qPCR. Each mRNA level was normalized to the β-actin mRNA level and expressed relative to the control (fold). Results are represented as means ± sd. *P < 0.05, **P < 0.01.
