# Supplementary figures and images for: TGF-β inhibitor treatment of H₂O₂-induced cystitis models provides biochemical mechanism for elucidating interstitial cystitis/painful bladder syndrome patients
Source: PLoS One. 2023 Nov 6;18(11):e0293983. doi: 10.1371/journal.pone.0293983 (PMC10627456; doi:10.1371/journal.pone.0293983)

**S3 Fig.**

**
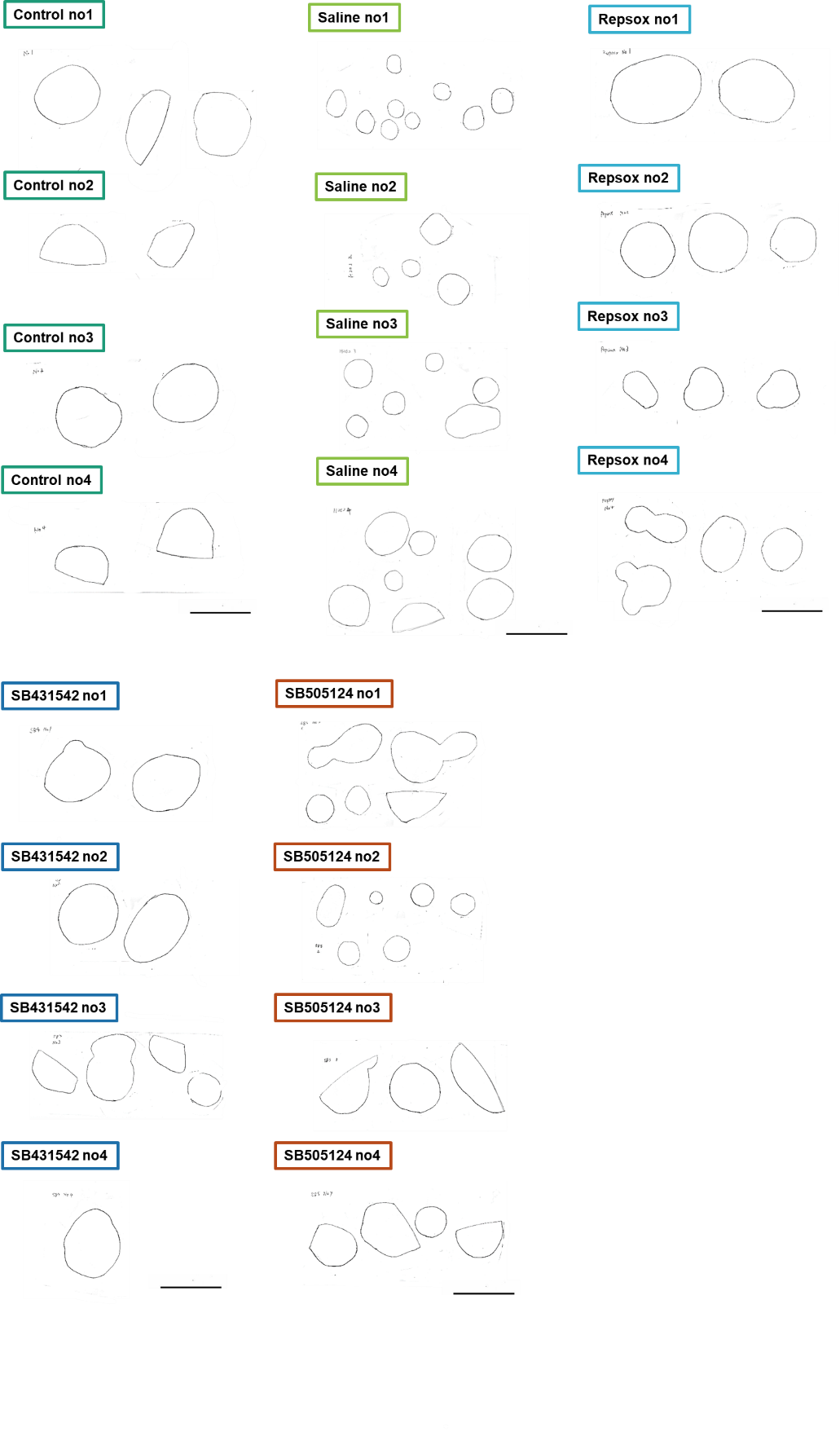
**

**S3 Fig. The stain sizes of each group.**

Scale bar = 50mm.

Supplement: S3 Fig — Scale bar = 50mm. (DOCX) [file pone.0293983.s003.docx]
