# Supplementary material for: TGF-β inhibitor treatment of H₂O₂-induced cystitis models provides biochemical mechanism for elucidating interstitial cystitis/painful bladder syndrome patients
Source: PLoS One. 2023 Nov 6;18(11):e0293983. doi: 10.1371/journal.pone.0293983 (PMC10627456; doi:10.1371/journal.pone.0293983)
Supplement: S4 Fig — The raw reads count of mRNAs related to barrier function in control, H₂O₂/saline-treated and H₂O₂/SB431542-treated groups (respectively n = 3) were measured by RNA-seq analyses. Results are represented as means ± sd. No statistically significant differences are shown because raw reads count is used. (DOCX) [file pone.0293983.s004.docx]

**S4 Fig.**

**
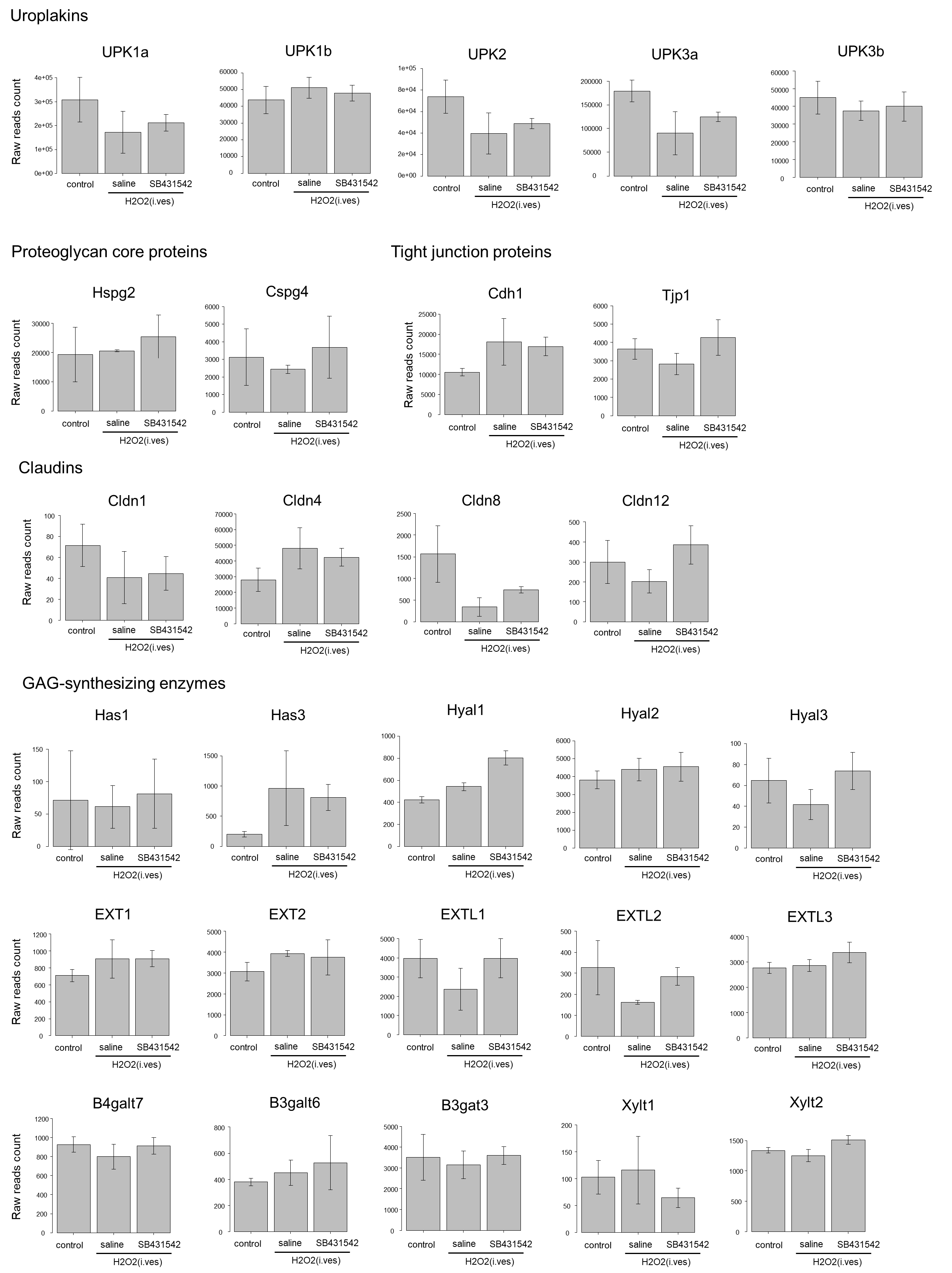
**

**S4 Fig. Expression of mRNAs related to barrier function is shown.** The raw reads count of mRNAs related to barrier function in control, H₂O₂/saline-treated and H₂O₂/SB431542-treated groups (respectively n=3) were measured by RNA-seq analyses. Results are represented as means ± sd. No statistically significant differences are shown because raw reads count is used.
