# Supplementary material for: TGF-β inhibitor treatment of H₂O₂-induced cystitis models provides biochemical mechanism for elucidating interstitial cystitis/painful bladder syndrome patients
Source: PLoS One. 2023 Nov 6;18(11):e0293983. doi: 10.1371/journal.pone.0293983 (PMC10627456; doi:10.1371/journal.pone.0293983)
Supplement: S5 Fig — Blood samples were collected for the assessment of liver and renal toxicity. sham, H₂O₂: intravesical H₂O₂ injection+ intraperitoneal saline injection, SB431542: intravesical H₂O₂ injection+ intraperitoneal SB431542 injection, respectively n = 4. Results are represented as means ± sd. *P<0.05. AST, aspartate aminotransferase; ALT, alanine aminotransferase; BUN, blood urea nitrogen; Cre, creatinine. (DOCX) [file pone.0293983.s005.docx]

**S5 Fig.**

**
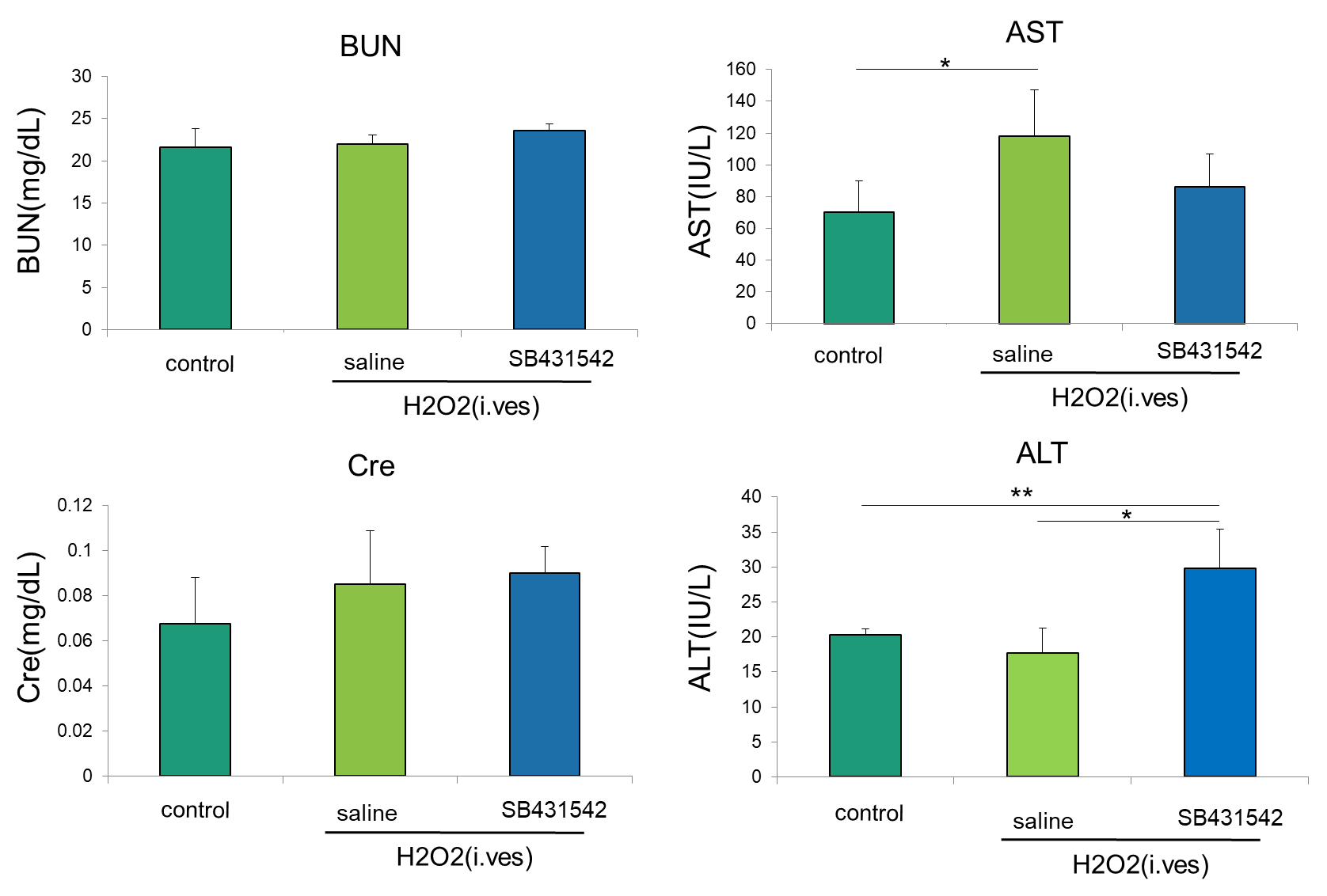
**

**S5 Fig. Biological toxicity in liver and kidney is not confirmed.**

Blood samples were collected for the assessment of liver and renal toxicity. sham, H₂O₂: intravesical H₂O₂ injection+ intraperitoneal saline injection, SB431542: intravesical H₂O₂ injection+ intraperitoneal SB431542 injection, respectively n=4. Results are represented as means ± sd. *P<0.05. AST, aspartate aminotransferase; ALT, alanine aminotransferase; BUN, blood urea nitrogen; Cre, creatinine.
