# Supplementary material for: TGF-β inhibitor treatment of H₂O₂-induced cystitis models provides biochemical mechanism for elucidating interstitial cystitis/painful bladder syndrome patients
Source: PLoS One. 2023 Nov 6;18(11):e0293983. doi: 10.1371/journal.pone.0293983 (PMC10627456; doi:10.1371/journal.pone.0293983)
Supplement: S6 Fig — Bladder weight (mg) on post-instillation day 10. sham, H₂O₂: intravesical H₂O₂ injection+ intraperitoneal saline injection, inhibitors(Repsox, SB431542, and SB505124): intravesical H₂O₂ injection+ intraperitoneal inhibitors injection, respectively n = 4. Chronological changes in whole body weight(g) on baseline, H₂O₂ pre-instillation day 0 and post-instillation day 4, 7. Results are represented as means ± sd. N.S.: no significant difference. (DOCX) [file pone.0293983.s006.docx]

**S6 Fig.**

**
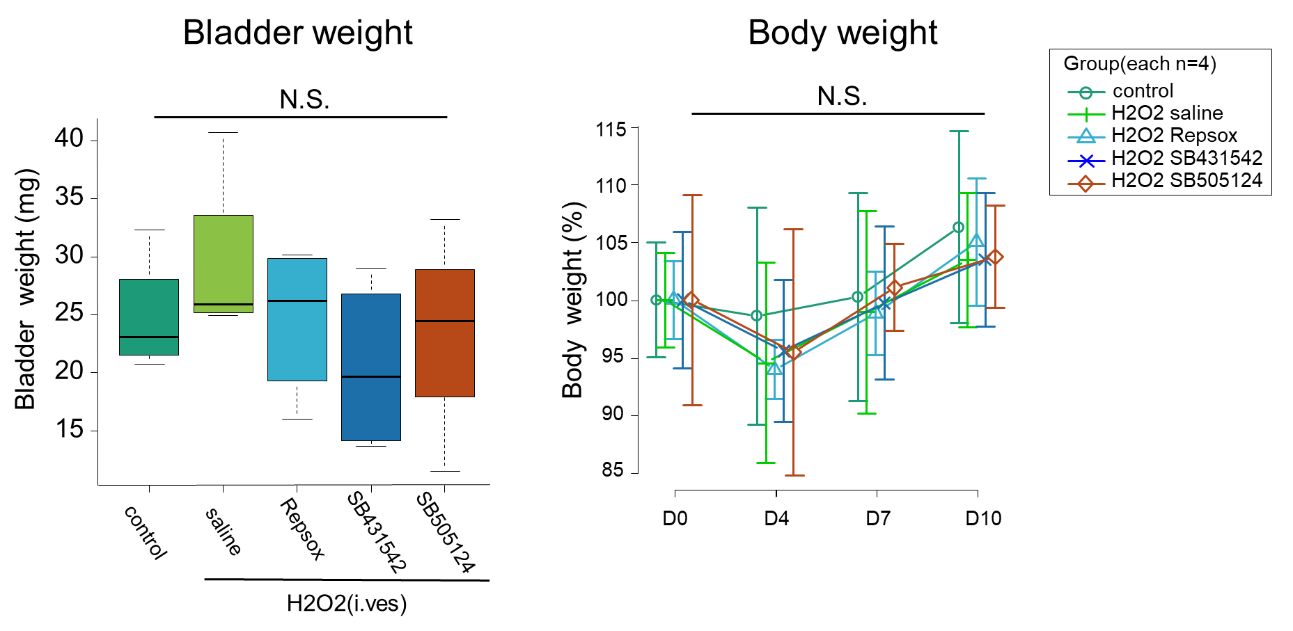
**

**S6 Fig. Body weight and bladder weight**

Bladder weight (mg) on post-instillation day 10. sham, H₂O₂: intravesical H₂O₂ injection+ intraperitoneal saline injection, inhibitors(Repsox, SB431542, and SB505124): intravesical H₂O₂ injection+ intraperitoneal inhibitors injection, respectively n=4. Chronological changes in whole body weight(g) on baseline, H₂O₂ pre-instillation day 0 and post-instillation day 4, 7. Results are represented as means ± sd. N.S.: no significant difference.
