# Supplementary material for: TGF-β inhibitor treatment of H₂O₂-induced cystitis models provides biochemical mechanism for elucidating interstitial cystitis/painful bladder syndrome patients
Source: PLoS One. 2023 Nov 6;18(11):e0293983. doi: 10.1371/journal.pone.0293983 (PMC10627456; doi:10.1371/journal.pone.0293983)
Supplement: Attachment — Submitted filename: Response to Reviewers.docx [file pone.0293983.s007.docx]

September, 2023

PLOS ONE, Academic Editor Yung-Hsiang Chen, Ph.D.

Manuscript No.: PONE-D-23-23604

Title: TGF-β inhibitor treatment of H₂O₂-induced cystitis models provides biochemical mechanism for elucidating interstitial cystitis/painful bladder syndrome patients.

Dear Prof. Yung-Hsiang Chen

Thank you for reviewing our above-referenced manuscript. We appreciate the valuable comments from the editorials and reviewers. We sincerely appreciate this opportunity to address the reviewers’ constructive criticisms and to revise our manuscript accordingly. The specific comments of the reviewers have been addressed in “Response to Reviewers” below.

Certainly, if there are any other questions, please do not hesitate to contact us.

Best regards

Hideto Taga, M.D. and Osam Mazda, M.D., PhD.

_________________________________________________________

**"Response to Reviewers ".**

●***Reviewer #1 (Remarks to the Author):*** 

**Question1**

***This is a well done and comprehensive study. The needed changes are relatively minor. The strengths are several and the weaknesses are mostly in the data and manuscript presentation. Strengths include the model itself. Other models of bladder inflammation including protamine, dilute HCl, and cyclophosphamide are as mentioned resolved within a week. The analysis of micturation patterns and gene expression add strength.***

**Answer to the Question1**

We appreciate the reviewer’s comment.

**Question2**

***Changes that need to be made are as follows. The material at the top of page 12 needs to be in the materials and methods.***

**Answer to the Question2**

We have taken into account the reviewer's feedback and opted to exclude the sentence in **Results** and added comments in **Materials and Methods:**

**Results (page 12, lines 6-9)**

~~We first examined a H₂O₂-induced cystitis model that we prepared based on previous reports with slight modifications.[3] Urinary frequency and one-time voiding volume were measured on days 1 and 8 to evaluate the effect of the H₂O₂ irritation on urinary function.~~

**Materials and Methods (page 7, lines 9-11):**

Urinary frequency and one-time voiding volume were measured on days 1 and 8 to evaluate the effect of the H₂O₂ irritation on urinary function.

**Question3**

***Figure S3 would be very helpful in the manuscript rather than in supporting material.***

**Answer to the Question3**

According to the reviewer #1’s comment, we have rephrased Figure S3 to Figure 2 in the Results section. Therefore, the figure numbers have been repositioned.

**Question4**

***However, a wealth of information on gene expression should be presented. These include genes involved with bladder barrier function such as uroplakins, proteoglycan core proteins identified previously as being involved in IC/BPS patients, tight junction proteins, claudins and GAG-synthesizing enzymes.***

**Answer to the Question4**

We have made bar graph with control, H₂O₂/saline-treated and H₂O₂/SB431542-treated groups by RNA-seq analyses in **Supporting information (page 35, lines10-14)** and added comments in **Discussion (page 26-27)** and **References (page 26-27)**:

**Supporting information (page 35, lines10-14)**

**S4 Fig. Expression of mRNAs related to barrier function is shown.**The raw reads count of mRNAs related to barrier function in control, H₂O₂/saline-treated and H₂O₂/SB431542-treated groups (respectively n=3) were measured by RNA-seq analyses. Results are represented as means ± sd. No statistically significant differences are shown because raw reads count is used.

**Discussion (page 26, line18	page 27, lines1-12)**

According to previous reports, proteins involved in urothelial barrier function include uroplakins, proteoglycan core proteins, tight junction proteins, and claudins.[1, 41] Uroplakin knockout mice have been used as mouse models of IC/PBS.[41] S4 Fig shows that the mean raw reads count of H₂O₂-treated mice was lower than that of control mice and SB43 mice, except for *Upk1b*. The mean raw reads count of *UPK3a* in H₂O₂-treated mice was lower than that of control mice. The mean raw reads count of *Cldn8* in SB43 mice was higher than that in H₂O₂-treated mice. No obvious differences were observed for proteoglycan core proteins and tight junction proteins. There were no previous reports on IC/PBS for GAG-synthesizing enzymes, but differences were found for *Hyal1* and *Xylt2*. ~~The detail mechanism by which TGF-β inhibitors protect the urothelial cell damage is still unknown.~~

**References (page 33, lines20-25)**

41.	Akiyama Y, Luo Y, Hanno PM, Maeda D, Homma Y. Interstitial cystitis/bladder pain syndrome: The evolving landscape, animal models and future perspectives. Int J Urol. 2020; 27(6): 491-503. https://doi.org/10.1111/iju.14229. PMID: 32246572.

●***Reviewer #2 (Remarks to the Author):***

**Question1**

***The study rationale was sound. The study design was well designed. The experiments were well conducted. The results were well explained.***

**Answer to the Question1**

We appreciate the reviewer’s comment.

**Question2**

***Some concerns existed on Fig. 4 H2O2 infusion augmented expression of Trpa1, IL-1β, Col7a1, Krt13, and Krt6a genes in the bladder, which was reversed by SB431542 administration. Although the genes mentioned above were reversed by TGF-beta inhibitors, whether these were the mediators of H2O2-induced cystitis remains elusive. The reasons need to be explained with caution.***

**Answer to the Question2**

We understand the reviewer’s concern. We have added comments in Discussion and References.

**Discussion (page 22, lines16-18)**

Oyama et al. reported that mRNA expression of *Trpa1* in bladders of the H₂O₂-treated mice was significantly higher than that in the control mice.[17]

**Discussion (page 24, lines3-5)**

Minami et al. reported that mRNA expression of *IL-1β* in bladders of the H₂O₂-treated mice was significantly higher than that in the control mice.[29]

**Discussion (page 25, lines10-14 )**

Although *Col7a1* has not been reported as a mediator of H₂O₂-induced cystitis, the results of increased *Col7a1* expression in H₂O₂-induced cystitis mice are consistent because the bladder wall of H₂O₂-induced cystitis mice has been shown to have increased fibrosis.[29]

**Discussion (page 26, lines11-18)**

*Krt6* and *Krt13* has not been reported as a mediator of H₂O₂-induced cystitis. The *Krt6* expression is seen in stratified epithelial cells featuring hyperproliferation, such as psoriasis. *Krt13* is associated with epithelial cell differentiation and signal transduction. [40] H₂O₂ destroys urothelial cells, followed by regeneration of urothelial cells through a process of proliferation and differentiation. Therefore, it seems that these increases in mRNA expression of keratins in H₂O₂-induced cystitis mice are consistent.

**References (page 31,32,33)**

**17.**	Oyama S, Dogishi K, Kodera M, Kakae M, Nagayasu K, Shirakawa H, et al. Pathophysiological Role of Transient Receptor Potential Ankyrin 1 in a Mouse Long-Lasting Cystitis Model Induced by an Intravesical Injection of Hydrogen Peroxide. Front Physiol. 2017; 8: 877. https://doi.org/10.3389/fphys.2017.00877. PMID: 29249972.

**29.**	Minami A, Tanaka T, Otoshi T, Kuratsukuri K, Nakatani T. Hyperbaric oxygen significantly improves frequent urination, hyperalgesia, and tissue damage in a mouse long-lasting cystitis model induced by an intravesical instillation of hydrogen peroxide. Neurourol Urodyn. 2019; 38(1): 97-106. https://doi.org/10.1002/nau.23822. PMID: 30411813

**40.**	Dmello C, Srivastava SS, Tiwari R, Chaudhari PR, Sawant S, Vaidya MM. Multifaceted role of keratins in epithelial cell differentiation and transformation. J Biosci. 2019; 44(2). PMID: 31180046.

**Question3**

***Similar situations happened in Fig. 5***

**Answer to the Question3**

We understand the reviewer’s concern. We have added comments in Discussion.

**Discussion (page 24, lines17,18	page 25, line1)**

Minami et al. showed that fibrosis area in bladder submucosa of the H₂O₂-treated mice increased than that of the control mice. [29]
